# Assessing Delays in Time to Diagnosis of Duchenne Muscular Dystrophy: A Survey of Current Primary Care Practices

**DOI:** 10.7759/cureus.99306

**Published:** 2025-12-15

**Authors:** Aravindhan Veerapandiyan, Joseph F Hagan, Paul Lipkin, Ruthwik Duvuru, Jaspreet Chahal, Jasleen K Chahal, Melissa Glasner

**Affiliations:** 1 Child Neurology, University of Arkansas for Medical Sciences, Little Rock, USA; 2 Pediatrics, The University of Vermont Children's Hospital, Burlington, USA; 3 Neurodevelopmental Pediatrics, Kennedy Krieger Institute, Baltimore, USA; 4 Project Management, University of Kentucky, Lexington, USA; 5 Outcomes Department, The France Foundation, Old Lyme, USA; 6 Medical Education Department, The France Foundation, Old Lyme, USA

**Keywords:** developmental pediatrics, diagnosis, duchenne, duchenne muscular dystrophy, dystrophinopathy

## Abstract

Background: Duchenne muscular dystrophy (DMD) is a progressive neuromuscular disorder in which delayed recognition remains a significant challenge in primary care. Early identification remains a significant challenge in primary care. Early identification is essential to enable timely referral, diagnostic testing, and initiation of supportive and disease-modifying therapies. However, variability in developmental screening practices and uncertainty regarding appropriate diagnostic evaluation may contribute to ongoing delays.

Aim: To investigate current practices in diagnosing and managing DMD, focusing on screening tools for developmental milestones and the referral process for suspected cases, and to understand the challenges and opportunities in early identification, diagnosis, and long-term care for individuals with DMD.

Methods: A 13-item needs survey was developed by a multidisciplinary team, including pediatricians and a pediatric neuromuscular specialist, to ensure content relevance and clarity. The face and content validity of the survey were established through expert review before distribution. This survey was then distributed nationally to 325 healthcare professionals using a mixed approach of open and closed-ended questions to evaluate screening tools used for developmental milestones, referral processes for suspected DMD diagnosis, and their involvement in ongoing DMD management.

Results: A total of 90% of clinicians were aware of checking creatinine kinase levels for DMD diagnosis; however, only 17% used it as a primary action for suspected motor delays. Approximately one-third of respondents did not use a primary screening tool for developmental delays, often opting for a "wait and see" approach instead. Thematic analysis of open-ended responses highlighted four main roles perceived by clinicians in DMD management: early detection, care coordination, referrals, and supporting patients and caregivers.

Conclusions: Clinicians play a crucial role in coordinating care for DMD patients, but there are gaps in standardized screening and diagnosis practices. Improved education for primary care clinicians is necessary to enhance the recognition and management of neuromuscular disorders, facilitating early access to therapies, and improving patient outcomes.

## Introduction

Duchenne muscular dystrophy (DMD) is a rare and severe genetic disorder affecting approximately one in every 3,500 male births globally [1]. DMD is characterized by progressive loss of muscle leading to the deterioration of skeletal, heart, and lung muscles, causing early mortality [1]. Early signs of DMD include delayed motor milestones, muscle weakness, calf hypertrophy, and difficulty running and climbing stairs [2,3]. As the disease progresses, individuals with DMD experience progressive muscle degeneration, leading to loss of ambulation by their early teens and complications such as cardiomyopathy and respiratory insufficiency [1-3]. Pediatricians, family medicine physicians, nurse practitioners, physician assistants, developmental-behavioral pediatricians, pediatric neurologists, physical and occupational therapists, and genetic counselors are some of the healthcare professionals involved in the primary care management of young children, along with performing developmental testing and resulting diagnostics.

Despite the availability of clinical lab tests and genetic testing, there is an average 2.5-year delay between the onset of symptoms and the time to diagnosis for patients with DMD [3,4]. Both parents/caregivers and healthcare practitioners do not readily associate these signs and symptoms with DMD, contributing to the diagnostic delay [3,4]. In the 2009 MD STARnet (Muscular Dystrophy Surveillance, Tracking, and Research Network) study, only 35% of patients ultimately diagnosed with DMD underwent creatine kinase (CK) testing during their first evaluation by a clinician (neurologists, orthopedists, child neurologists, neuromuscular specialists, and developmental pediatricians) [4]. Meanwhile, delays in obtaining CK testing prolonged the diagnosis of DMD in this cohort [4]. In 2013, the American Academy of Pediatrics (AAP) created a clinical report on screening children with motor delay when CK screening was promoted for children with motor delay. This was again reiterated in the developmental screening clinical report of 2020. Despite these efforts, the 2022 MD STARnet survey suggests no impact [5-7].

In the FOR-DMD (Finding the Optimum Regimen for Duchenne Muscular Dystrophy) study, the investigators observed that parents, on average, voiced their concerns at 29.8 months, citing motor delays, walking difficulties, and speech delays as common symptoms [6,8]. The group also found a mean diagnostic delay of 25.9 months before genetic diagnosis at 53.9 months [6]. Notably, following incidentally elevated CK levels, the mean diagnostic delay was shortened to 6.4 months [6].

The present exploratory study aimed to examine the current practices in the diagnosis and management of DMD and the referral process for suspected DMD diagnosis. By addressing these key areas, the study aimed to provide a comprehensive understanding of the challenges and opportunities for optimizing early identification, diagnosis, and management for individuals with DMD.

## Materials and methods

An internet-based survey was distributed to 325 pediatricians and family practitioners across the US. Clinicians were selected based on their clinical focus and sent emails using a purchased contact list from an email marketing distributor. The survey was distributed through an email marketing company, with reminders sent two weeks after the initial email. A mixed approach comprising both open and closed-ended questions was used to assess the screening tools used for developmental milestones, the referral process for suspected DMD diagnosis, and their role in ongoing management practices for patients with DMD.

Three authors developed the survey with two pediatricians and a pediatric neuromuscular neurologist, who reviewed and provided feedback. The 13-item survey (Appendix) was organized into four sections: demographic information, identifying knowledge gaps regarding DMD (e.g., signs & symptoms and testing), current practices in DMD management (e.g., screening tools used and referral process), and educational preferences (e.g., the format of education and appropriate length).

Skip logic was used in the survey format to streamline questions regarding current practices. When asked, “What is your primary course of action if you see a child with motor delay at an 18-month well-child visit?”, if the respondent chose “watch and wait,” they were directed to the question, “How long do you generally wait before a re-check?” However, if they responded, “perform further labs and testing,” they received the follow-up question, “When you perform additional labs or tests because of motor delay, which type is most common?” All respondents received questions on demographics, DMD knowledge, and educational preferences.

Results were analyzed using a mixed methods approach. Quantitative analysis was conducted using frequencies to assess DMD knowledge, early diagnosis, and screening practices, as well as the referral process. Qualitative data were collected to better understand clinicians’ perceptions of their role in DMD management. Responses from de-identified open-ended questions were reviewed by the authors and assessed for themes. Analysts did not have access to respondents' demographics or quantitative survey responses during the initial coding phase. Authors independently coded the responses, met to reconcile differences, and reached consensus on the final themes to minimize interpretive bias.

## Results

A total of 113 of 325 providers responded, and 85 (75.2%) completed the survey, with the other 28 (24.8%) partially completing it. Of the 113 respondents, 85% were clinicians, including physicians (71%, n = 77), nurse practitioners (10%, n = 11), and physician associates (4%, n = 4), and 15% were other care providers. Of 103 participants who answered this demographic question, 19% (n = 20) identified as family practitioners, 24% (n = 25) as internal medicine-pediatrics, 35% (n = 36) were pediatricians, and 49% (n = 50) of clinicians worked in community practices not affiliated with medical centers.

Almost one-third of respondents reported not using a primary screening tool to detect developmental delays at well-child visits (Table 1). Even though most respondents (90%, n = 78) were aware of checking CK levels as an initial test to diagnose DMD, clinicians often still selected a “watch and wait” approach versus using a formal tool to confirm suspected motor delays.

**Table 1:** Assessment tools used at well-child checks.

Assessment tools (Check all that apply)	18 months (%)	24 months (%)	30 months (%)
Ages and Stages Questionnaire (ASQ) [9]	33 (40.2%)	33 (42.9%)	36 (48.0%)
Pediatric Evaluation of Development Status (PEDS or PEDS-DM) [10]	20 (24.4%)	24 (31.2%)	22 (29.3%)
Survey of Well Being in Youth and Children (SWYC) [11]	8 (9.8%)	9 (11.7%)	10 (13.3%)
Infant and Toddler Development Checklist [12]	20 (24.4%)	16 (20.8%)	12 (16.0%)
Modified Checklist for Autism in Toddlers (M-CHAT or M-CHAT-R/F) [13]	29 (35.4%)	25 (32.5%)	16 (21.3%)
Other (Milestone Tracker App)	2 (2.4%)	2 (2.6%)	1 (1.3%)
I do not use a formal tool	24 (29.3%)	21 (27.3%)	24 (32.0%)

Only 17% selected performing further labs and testing, such as a CK test, as a primary course of action when suspecting motor delays at 18 months (Table 2). Additionally, testing percentages remained consistently low across patient ages, with only 18% of clinicians testing CK levels at 24 months and 15% testing at 30 months. By selecting an alternate primary course of action in Table 2, over 80% (n = 38) of respondents were waiting to perform further labs and testing, including checking for a CK level when motor delay was suspected during a well-child visit.

**Table 2:** Primary course of action for a child with suspected motor delay at a wellness check.

Primary course of action	18 months (%)	24 months (%)	30 months (%)
Watch and wait	3 (4.0%)	1 (1.3%)	2 (2.7%)
Refer to a pediatric specialist	28 (36.8%)	39 (51.3%)	45 (60.0%)
Refer a child for physical therapy or evaluation	29 (38.2%)	18 (23.7%)	14 (18.6%)
Perform further labs and testing	13 (17.1%)	14 (18.4%)	11 (14.7%)
Other (Depends on the severity of the delay and how delayed)	3 (3.9%)	4 (5.3%)	3 (4.0%)

To gain a better understanding of screening procedures and referral processes, open-ended responses were collected on clinicians’ perceptions of their role in DMD management for their patients. Four themes emerged in a thematic analysis: early detection, referrals, care coordination, and supporting patients as well as caregivers (Table 3). The first theme indicated participants felt their role focused on early diagnosis, “providing prenatal diagnosis and genetic counseling.” Clinicians also expressed their role in making appropriate and timely referrals and providing follow-up care. The emphasis on follow-up care was also seen in a theme that stressed the importance of care coordination or “coordinating specialty care and managing overall wellness,” while also “being sure to do follow-ups and making sure that treatments are followed” for DMD management.

**Table 3:** Thematic analysis of clinicians' perceptions of their role in DMD management. DMD: Duchenne muscular dystrophy.

Role category	Perceived roles in DMD management
Early detection	“Provide genetic counseling and aid in the testing and diagnosis process.” “Diagnosing and treating.” “Providing prenatal diagnosis and genetic counseling.”
Timely referrals	“Place referrals and follow up that the referral was done.” “Referrals to evaluate for pulmonary issues like lung restriction.” “Sending appropriate referrals.”
Care coordination	“Coordinating specialty care and managing overall wellness.” “Function more as one who does an early diagnosis, refers, and translates the management to the family.” “Being sure to do follow-ups and making sure that treatments are followed.”
Supporting patients and caregivers	“Keep the family informed while the patient is being primarily managed by the pediatric specialist.” “Helping with services at school, support for equipment needs, etc.” “Support and counsel the family and patient.”

The final theme that emerged illustrated that many practitioners explained that their role was centered on supporting their patients and caregivers. One participant said, “Keep the family informed while the patient is being primarily managed by the pediatric specialist.” While another clinician also stated that “helping with services at school, support for equipment needs, etc.,” was part of their role.

## Discussion

Collectively, these results showed that clinicians perceived their primary role as providing support for the patient and family throughout the care coordination of DMD management. This exploratory study emphasizes the importance of improving awareness and the use of assessment tools, such as CK testing, by clinicians as a standard practice for well-child visits to diagnose patients earlier.

Effectively addressing the multifaceted aspects of DMD requires a systematic, standardized, and well-organized multidisciplinary approach [1]. Clinicians play a crucial role in coordinating specialized care delivery by fostering collaboration among healthcare professionals to address the intricate needs of patients [14]. The Centers for Disease Control’s "Learn the Signs. Act Early" (LTSAE) program is a comprehensive initiative aimed at improving early identification of developmental delays and disabilities, including autism, in children from birth to five years old [15]. The program aims to reduce the "wait and see" approach by placing milestones at ages when at least 75% of children would be expected to exhibit them, making even one missing milestone more actionable [15]. The AAP initiatives of 2013 and 2020 aimed to get children with motor delay screened for DMD with CK when a delay is identified [5,6]. However, the LTSAE does not specifically address early identification of muscular dystrophy and other neuromuscular or neuromotor disorders. Despite these efforts, little was known by respondents regarding current DMD screening and diagnostic practices [4]. In this exploratory study, primary care practitioners were knowledgeable about DMD signs, symptoms, and testing. However, self-reported practice data showed a delay in using assessments to improve timely DMD diagnosis and referrals [5-7].

Caregivers and school personnel are most often the first to notice signs and symptoms of DMD in children [16]. Subsequently, the initial healthcare practitioner consulted for concerns about DMD signs and symptoms is often a pediatric or family medicine practitioner [4]. The most frequently used screening tools across patients aged 18 through 30 months upon clinical suspicion of a motor delay included the Ages and Stages Questionnaire (ASQ) [9], Pediatric Evaluation of Development Status (PEDS or PEDS-DM) [10], Modified Checklist for Autism in Toddlers (M-CHAT or M-CHAT-R/F) [13], and the Infant and Toddler Development Checklist [15]. However, nearly one-third of respondents reported not using a primary screening tool to detect developmental delays at well-child visits, despite AAP's efforts recommending such practices [5,6]. Early screening for DMD is crucial for timely intervention and management [16,17]. Elevated serum creatine kinase indicates muscle damage and can serve as a sensitive biomarker for an early screening tool for children with DMD [16,17]. It can also prompt referral to a neurologist or neuromuscular specialist for further evaluation [16,17]. Additionally, genetic testing can confirm the diagnosis before symptoms fully manifest [17].

Delays in diagnosis reflect a large clinical gap in practice that has not been ameliorated since initially identified in 2009, and the AAP reports of 2013 and 2020 [5-7]. This implies a need for a more standardized approach for the screening tools used at wellness checks for patients with suspected motor delays. Albeit variable, DMD also impacts the heart, leading to cardiomyopathy and other cardiac complications [18,19]. Shin et al. reported an increased proportion of patients under the age of 10 years receiving angiotensin-converting enzyme inhibitors (ACEi) annually, and therefore, delayed diagnosis also means that cardiac issues may not be addressed promptly, increasing the risk of serious heart problems and reducing life expectancy [19]. A recent report on multicenter DMD registry data reported improved overall survival and decreased hospitalization rates due to heart failure with prophylactic administration of ACEi. DMD can also affect the respiratory muscles, leading to breathing difficulties and respiratory infections [20,21]. Delayed diagnosis may result in inadequate management of respiratory complications, increasing the risk of respiratory failure and pneumonia [20,21].

Early detection and diagnosis of DMD are crucial, as treatments are most effective when administered early in the disease process [3,22]. Early diagnosis permits early initiation of supportive therapies, such as physical therapy and corticosteroids, which can help delay disease progression and improve quality of life [16,17]. Furthermore, early identification facilitates access to emerging treatments like gene therapy and exon skipping drugs that hold promise in addressing the underlying genetic defects [4]. Additionally, early detection can also enable patient participation in research and access to clinical trials for emerging DMD therapies that may modify its natural course [22]. Therefore, pediatricians and family practitioners require additional education and training on recognizing, screening, and managing patients with neuromuscular disorders. Primary care clinicians can play a critical role in the early diagnosis of DMD. It is important for clinicians to stay informed and be vigilant about screening for delayed motor and developmental milestones. Although many primary care clinicians knew that they should order a CK test as part of the initial evaluation for any child presenting with motor delays or weakness, its underuse may reflect barriers beyond awareness, including workflow limitations, competing clinical demands, or uncertainty about when testing is warranted [5-7]. Primary care clinicians also play a key role in the ongoing care and management of individuals with DMD, working closely with the multidisciplinary care team. This includes monitoring for and managing the various medical complications associated with DMD, such as cardiac, respiratory, and orthopedic issues. Finally, primary care clinicians should be aware of the latest treatment options and work closely with the family and the healthcare and treatment intervention teams to ensure timely initiation of appropriate therapies.

In the future, we could leverage technology and artificial intelligence (AI ), such as AI-assisted symptom tracking that analyzes movement patterns and flags early DMD signs, as well as flagging of boys with persistently elevated CK or motor delays on electronic health records for neuromuscular evaluation. Technology, along with the empowerment of parents and caregivers through public awareness campaigns and streamlined referral pathways, may help diagnose and manage DMD.

Finally, our study has several strengths, such as the national scope of participating clinicians, a mixed methods study design, and expert-informed survey development. However, limitations exist, such as a modest overall response rate of 34.8%, which may introduce nonresponse bias and limit generalizability. As with all self-report surveys, responses may reflect recall bias or social desirability bias. Additionally, while open-ended responses underwent independent review by analysts, thematic coding may still carry interpretive subjectivity. Finally, although the study was deemed exempt by the University of Arkansas for Medical Sciences (UAMS) Institutional Review Board (IRB), the use of an anonymous online survey limits the ability to verify individual respondent characteristics. Therefore, these limitations should be considered alongside the study's strengths when interpreting its implications for early DMD detection and primary care practices.

## Conclusions

Our study highlights persistent gaps in the early recognition and diagnostic workup of DMD, within primary care settings, despite longstanding recommendations and widespread clinician awareness of CK testing as a critical first step. Although clinicians recognize their important roles in early detection, care coordination, and family support, the underuse of standardized development screening tools and reliance on delayed evaluation strategies continue to impede timely diagnosis.

Strengthening clinician education, integrating consistent screening practices, and developing streamlined pathways for early referral and testing are essential steps toward reducing diagnostic delays. As emerging therapies increasingly depend on early intervention, optimizing primary care practices remains central to improving outcomes and quality of life for individuals with DMD and their families.

## Appendices

**Table 4:** Duchenne muscular dystrophy (DMD) education for pediatricians and family practice providers: survey on knowledge, current practice, and educational needs. Survey instrument used to assess demographics, knowledge gaps, current clinical practices, and educational preferences among pediatric and family medicine healthcare providers regarding Duchenne muscular dystrophy (DMD). The table includes items evaluating provider awareness of early signs and symptoms of DMD, diagnostic approaches, referral patterns, developmental screening tool usage, management experience, and preferred formats for continuing medical education.

9190: DMD Education for Pediatricians and Family Practice
Demographics questions
1. What is your healthcare professional category?
A. Physician
B. Pharmacist
C. Physician Associate
D. Nurse Practitioner
E. Registered Nurse
F. Other____________________
2. What is your area of primary clinical focus?
A. Pediatrics
B. Family Medicine
C. Internal Medicine
D. Pediatric Neurology
E. Other _______________
3. Which best describes your practice?
A. Academic medical center
B. Community practice affiliated with an academic medical center
C. Community practice not affiliated with an academic medical center
D. Federally Qualified Health Center (FQHC)
4. If you answered B, C, or D: What is your practice’s distance from the nearest academic medical center?
A. ≤ 25 miles
B. 26-50 miles
C. 51-100 miles
D. ≥ 100 miles
Identification of Knowledge Gaps in Pediatrics/Family Medicine Regarding DMD
1. How would you rate your awareness of the early signs/symptoms of Duchenne muscular dystrophy (DMD)?
A. Not at all aware
B. Slightly aware
C. Somewhat aware
D. Moderately aware
E. Extremely aware
2. What percentage of males diagnosed with DMD have a family history of DMD?
A. 65-75%
B. 5-10%
C. 25-50%
D. 0-5%
3. Which of the following are early signs/symptoms of DMD common in patients 18 months to 30 months old? (Choose all correct answers)
• Motor delay
• Toe walking
• Autism spectrum disorder behaviors
• Seizure
• Muscle stiffness
• Breathing difficulties
4. Which of the following are signs/symptoms of DMD common in patients 4 to 5 years old? (Choose all that apply)
• Motor delay
• Gait abnormality
• Seizure
• Muscle stiffness
• Breathing difficulties
5. Which of the following screening/initial tests can help with diagnosing DMD?
• Creatine kinase levels
• Complete blood counts
• Liver function test
• Alkaline phosphatase levels
6. Which is/are true regarding DMD treatment? (Choose all correct answers)
• There are multiple treatments that slow down the progression of the disease
• There are treatments that target the genetic mechanism of DMD (i.e., exon skipping agents)
• There are no treatments that significantly alter the course of the disease
• There are multiple treatments that cure DMD
Current Practice
1. What “general” screening tool are you using to detect developmental delays at 18-month well-child visits? (Check all that apply)
• Ages and Stages Questionnaire (ASQ)
• Pediatric Evaluation of Development Status (PEDS or PEDS-DM)
• Survey of Well Being in Youth and Children (SWYC)
• Infant and Toddler Development Checklist
• Modified Checklist for Autism in Toddlers (M-CHAT or M-CHAT-R/F)
• I do not use a formal tool
• Other__________________
2. What “general” screening tool are you using to detect developmental delays at 24-month well-child visits? (Check all that apply)
• Ages and Stages Questionnaire (ASQ)
• Pediatric Evaluation of Development Status (PEDS or PEDS-DM)
• Survey of Well Being in Youth and Children (SWYC)
• Infant and Toddler Development Checklist
• Modified Checklist for Autism in Toddlers (M-CHAT or M-CHAT-R/F)
• I do not use a formal tool
• Other__________________
3. What “general” screening tool are you using to detect developmental delays at 30-month well-child visits? (Check all that apply)
• Ages and Stages Questionnaire (ASQ)
• Pediatric Evaluation of Development Status (PEDS or PEDS-DM)
• Survey of Well Being in Youth and Children (SWYC)
• Infant and Toddler Development Checklist
• Modified Checklist for Autism in Toddlers (M-CHAT or M-CHAT-R/F)
• I do not use a formal tool
• Other__________________
4. What is your primary course of action if you see a child with motor delay at an 18-month well-child visit?
A. Watch and wait (branches to Q6)
B. Refer to a pediatric specialist (branches to Q7)
C. Refer a child for physical therapy or evaluation (branches to Q8)
D. Perform further labs and testing (branches to Q9)
E. Other________________
5. What is your primary course of action if you see a child with motor delay at a 30-month well-child check?
A. Watch and wait (branches to Q6)
B. Refer to a pediatric specialist (branches to Q7)
C. Refer a child for physical therapy or evaluation (branches to Q8)
D. Perform further labs and testing (branches to Q9)
E. Other________________
BRANCHING
6. If choosing watch and wait, how long do you generally wait before a re-check?
A. 1 month
B. 3 months
C. 6 months
D. 1 year
E. Other______________________
7. When you refer patients to a pediatric specialist because of motor delay, which is most common?
A. Child Neurology
B. Developmental-Behavioral Pediatrics
C. Neurodevelopment
D. Physical Medicine and Rehab
E. Genetics
F. Other__________________________
8. When you refer patients to therapy because of motor delay, which is most common?
A. Physical Therapist
B. Occupational Therapist
C. Early Intervention Services
D. Other _______________________
9. When you perform additional labs or tests because of motor delay, which type is most common?
A. Genetic testing
B. Creatine kinase
C. Liver function tests
D. Other __________________________
10. Do you currently manage any patients with DMD?
A. Yes (Branch to Questions 11 & 12)
B. No (Continue to Question 13)
11. How many of your patients are being managed for DMD in your practice?
A. 1-5
B. 6-16
C. 11-15
D. 16-20
E. > 21
12. How did these patients first present in your clinic?
A. Parent identified concern
B. Well-child visit
C. Referral from another specialty
D. Other
13. What is and/or would be your role in managing patients who are diagnosed with DMD? (Open-ended response)
Educational preferences
1. Please select your preferred format for education on DMD.
• On-demand video/webinar
• On-demand audio (podcast)
• Interactive online module
• Social media (Twitter, Facebook)
• Online gaming activity
• Live virtual presentation/discussion
• Live in-person presentation/discussion
• Online text/journal articles
2. When completing continuing medical education activities, what is your preferred length?
A. Bundled microlearning activities equating 15 minutes (e.g., three, five-minute activities)
B. 15 minutes
C. 30 minutes
D. 45 minutes
E. 60 minutes
F. I don’t have a preference
